# The first mecp2-null zebrafish model shows altered motor behaviors

**DOI:** 10.3389/fncir.2013.00118

**Published:** 2013-07-16

**Authors:** Thomas Pietri, Angel-Carlos Roman, Nicolas Guyon, Sebastián A. Romano, Philip Washbourne, Cecilia B. Moens, Gonzalo G. de Polavieja, Germán Sumbre

**Affiliations:** ^1^Ecole Normale Supérieure, Institut de Biologie de l'ENSParis, France; ^2^Inserm, U1024Paris, France; ^3^CNRS, UMR 8197Paris, France; ^4^Instituto Cajal, Consejo Superior de Investigaciones CientíficasMadrid, Spain; ^5^Institute of Neuroscience, University of OregonEugene, OR, USA; ^6^Division of Basic Sciences, Fred Hutchinson Cancer Research CenterSeattle, WA, USA

**Keywords:** zebrafish, motor behavior, Rett syndrome, mecp2, early development, thigmotaxis

## Abstract

Rett syndrome (RTT) is an X-linked neurodevelopmental disorder and one of the most common causes of mental retardation in affected girls. Other symptoms include a rapid regression of motor and cognitive skills after an apparently early normal development. Sporadic mutations in the transcription factor MECP2 has been shown to be present in more than 90% of the patients and several models of MeCP2-deficient mice have been created to understand the role of this gene. These models have pointed toward alterations in the maintenance of the central nervous system rather than its development, in line with the late onset of the disease in humans. However, the exact functions of MeCP2 remain difficult to delineate and the animal models have yielded contradictory results. Here, we present the first *mecp2*-null allele mutation zebrafish model. Surprisingly and in contrast to MeCP2-null mouse models, *mecp2*-null zebrafish are viable and fertile. They present nonetheless clear behavioral alterations during their early development, including spontaneous and sensory-evoked motor anomalies, as well as defective thigmotaxis.

## Introduction

Epigenetic regulations of gene expression play a crucial role in brain development and maturation. Accordingly, alterations of factors involved in epigenetic regulation at the level of gene transcription lead to critical defects in brain function. Methyl-CpG-binding protein 2 (MECP2), a basic transcription factor, is a major epigenetic regulator. It was first described as a global repressor of transcription (Lewis et al., 1992). More recently, apart from its well documented repressor activity, MECP2 seems to act as a gene expression activator (Yasui et al., 2007; Chahrour et al., 2008), to modulate gene expression by long range chromatin remodeling (Skene et al., 2010) and to regulate RNA splicing (Young et al., 2005).

MeCP2, present in all vertebrate species, appears to be an innovation of this phylum. It shows a high level of conservation among mammals with, for instance, more than 95% protein sequence identity between human and mouse, and to a lesser extent, between humans and zebrafish (48%). In mammals, like in zebrafish, MeCP2 appears to be expressed throughout the body in multiple organs, and particularly enriched in the nervous system. Its expression in the nervous system is first detected during early embryonic development and it is highly expressed in postmitotic neurons (Shahbazian et al., 2002a; Curado et al., 2007). The structure and expression patterns of MeCP2 in zebrafish and mammals are similar (Coverdale et al., 2004), suggesting strong evolutionary pressure and therefore probable conserved functions.

Alterations of MECP2 functions have been shown to be causative of several neurodevelopmental diseases associated with autism spectrum disorders. In particular, sporadic mutations of MECP2 have been uncovered in more than 90% of patients diagnosed with Rett syndrome (RTT, Amir et al., 1999). At the clinical level, patients with RTT are asymptomatic during the first 6 to 18 months of life, appearing to develop normally. Subsequently, a period of stagnation precedes a net regression of the acquired skills. It is during this period that mental retardation appears and psychomotor skills degrade. Some patients also display seizures, abnormal cardiac, or breathing cycles (Matsuishi et al., 2011).

Various mouse models of RTT have been created, ranging from null-MeCP2 mutations to specific point ones mimicking those in humans. These models closely phenocopy several motor and cognitive dysfunctions described in RTT patients (Chen et al., 2001; Guy et al., 2001; Shahbazian et al., 2002b; Moretti et al., 2005, 2006; Picker et al., 2006; Santos et al., 2007). These different models point toward alterations of synaptic and neural circuits maturation and maintenance, in particular an imbalance between excitation and inhibition (Dani et al., 2005). Interestingly, this imbalance could affect various brain regions and at different stages of development (Blue et al., 2011; Kron et al., 2012). In addition to the effect on neurons, it has been recently shown that MeCP2 dysfunction may also perturb glial cells (Ballas et al., 2009; Lioy et al., 2011). Overall, these findings suggest that MeCP2 functions appear to be cell type and developmental stage dependent, thus impeding the delineation of the entire spectrum of MeCP2 functions and the association of a particular functional alteration to a specific phenotype.

Zebrafish has recently gained much attention as a vertebrate model for human neurodevelopmental and neurodegenerative diseases (Panula et al., 2006; Beattie et al., 2007; Morris, 2009; Brennan, 2011; Kabashi et al., 2011; Steenbergen et al., 2011; Xi et al., 2011). Importantly, zebrafish produce a large number of progeny, with fast and external embryonic and larval development. They have a large repertoire of well-studied motor behaviors (for recent review, see Saint-Amant, 2010). Molecular manipulations of its genome can be achieved through forward genetic screening of mutagenized fish (Draper et al., 2004) or genome editing methods (Huang et al., 2011). Overall, these features have established zebrafish as a powerful and complementary vertebrate model for behavioral and genetic analysis, and more recently as an invaluable human disease model.

Here, we introduce the first zebrafish model of RTT, in which mecp2 function was abolished. A kinematic analysis of motor activity in embryos and young larvae reveals alterations of spontaneous, sensory-evoked and thigmotaxic behaviors.

## Materials and methods

### Animals

AB adult, larva, and embryo zebrafish, from the University of Oregon Zebrafish Facility, were maintained at 28.5°C on a 14–10 h on/off light cycles at the Institut de Biologie de l'Ecole Normale Supérieure zebrafish facility. Embryos and larvae were grown accordingly to Westerfield (2000). All experimental procedures were performed at room temperature (21–23°C). Zebrafish *mecp2*^Q63*^ mutation was generated through *N*-ethyl-*N*-nitrosourea (ENU)-mutagenesis and selected by TILLING as previously described (Draper et al., 2004). As ENU-mutagenesis generates random mutations throughout the genome, heterozygote *mecp2* mutant fish were outcrossed several times in the AB background to remove off-target mutations. Mutant fish were identified by PCR using DNA extracted from fin clip (the primers 5′-AAAGGAAAGGCATGATGTGG-3′ and 5′-GTATCGCCAACCTTTTGGAA-3′ flank the position of the mutation), followed by sequencing. To keep the closest genetic background between different genotypes in the experiments, heterozygote *mecp2* mutant embryos and larvae were generated by outcrossing homozygote *mecp2* mutants with AB wild-type fish. Wild-type, heterozygote and homozygote *mecp2* mutant fish are, respectively, labeled WT, Het, and Mut in the figures. Morphological assessment of aged-matched wild-type, homozygote and heterozygote mecp2 mutant embryos and larvae did not reveal any difference, indicating that the rate of development is similar in the three groups of embryos and larvae. Accordingly, we used hours post fertilization (hpf) and days post fertilization (dpf) to stage embryos and larvae, respectively.

All procedures were carried out in compliance with the guidelines of the University of Oregon and Le Comite d'Ethique pour l'Experimentation Animale Charles Darwin.

### Synteny mapping

Synteny mapping was performed by comparing the organization of the human genomic region bearing the MECP2 gene, with the zebrafish genome. To carry out this analysis, BLASTp searches were implemented using human proteins, inferred from genes present in this region, against the zebrafish database. Each first hit in the searches where then confirmed by a reciprocal BLASTp search. Only the reciprocal best hits of BLASTp searches were considered.

### Western blotting

Adult brain was homogenized in 1× RIPA extraction buffer (Cell Signaling) complemented with protease inhibitor cocktail tablets (Roche) and incubated on ice for 45 min, then centrifuged at 14,000 g for 5 min. The supernatant was collected and the protein concentration was measured with the BCA Protein Assay Kit (Pierce). Samples were then boiled in Laemmli buffer, separated on SDS-PAGE gel, transferred to nitrocellulose membrane and successively probed with a chicken anti-human MeCP2 C-terminal antibody (gift from Dr. LaSalle) at a dilution of 1 μg/ml and visualized with a goat anti-chicken IgY HRP-coupled antibody (Jackson Laboratories) at a dilution of 0.1 μg/ml.

### Kinematic analysis of motor behaviors

Experiments were performed on 25 hpf, 51 hpf embryos, and 6 dpf larvae. The 25 hpf embryos were dechorionated at least one hour before the experiments. All embryos and larvae were acclimatized to room temperature for at least 20 min before the beginning of the experiments. To estimate the rate of spontaneous contractions, the freely moving 25 hpf embryos were recorded under a stereo-microscope (Leica LZMFIII) using a monochrome digital camera (PixeLINK PL-A741) at 20 Hz. over a period of 5 min. Fifty one hpf larvae were subjected to light mechanical stimulation using an eyelash either on the side of the trunk or on the head of the larvae. Stimulations were applied 5–7 times separated by at least 1 s. Larva motor responses were recorded at 100 Hz. The startle responses induced by the stimulations were analyzed with ImageJ software. Only the duration of complete coilings were measured. These were defined as the period between the first deviation of the trunk from its resting state until its return to its initial position. In case of multiple contractions after a stimulation, only the first coiling period was taken into account. To monitor the spontaneous swimming activity, 6 dpf wild-type and mecp2-mutant larvae were randomly placed in a custom-made Plexiglas 35-well plate (15 mm diameter × 0.5 mm height) filled-in with 500 μ l embryo medium at room temperature and let to habituate for 20 min before the experiment. Homogeneous illumination was provided by an electroluminescent panel (MiniNeon, France). Spontaneous motor behavior was monitored with a ImagingSource DMK 21BF04 camera at 30 Hz for 15 min. Custom Matlab scripts (MathWorks) were developed to compute the trajectories, activity, velocities, and the thigmotactic behavior. All Matlab scripts are available upon request. Larva activity was defined as the percentage of time in which movement was detected. Activity bouts and resting times were calculated as the periods of movement and between consecutive movements, respectively. Both were fitted to a power law of the *Y* = *a*X^−k^ form. Thigmotactic behavior analysis described the relative position of the larvae within the well over time. For this purpose, we divided the wells in two zones: an inner zone at the center of the well covering 36% of the total area, and an outer ring region covering the remaining 64% (see Figure 3B). To take into account potential alterations of motor functions in mecp2-null larvae, we analyzed the kinematics of a subset of swimming periods. Criteria for the selection of these subsets of trajectories are detailed in the results section.

### Statistics

Results are presented by classical box-and-whisker diagrams with the median as the central red mark, the first quartile (q1) and third quartile (q3) for the edge of the box, and the extreme data points represented by the whiskers in the range of q1–1.5^*^(q3–q1) to q3 + 1.5^*^(q3–q1). Statistical significance was assessed using the Kolmogorov–Smirnov test.

## Results

### Zebrafish mecp2 is an ortholog of human mecp2

**Figure 1:**
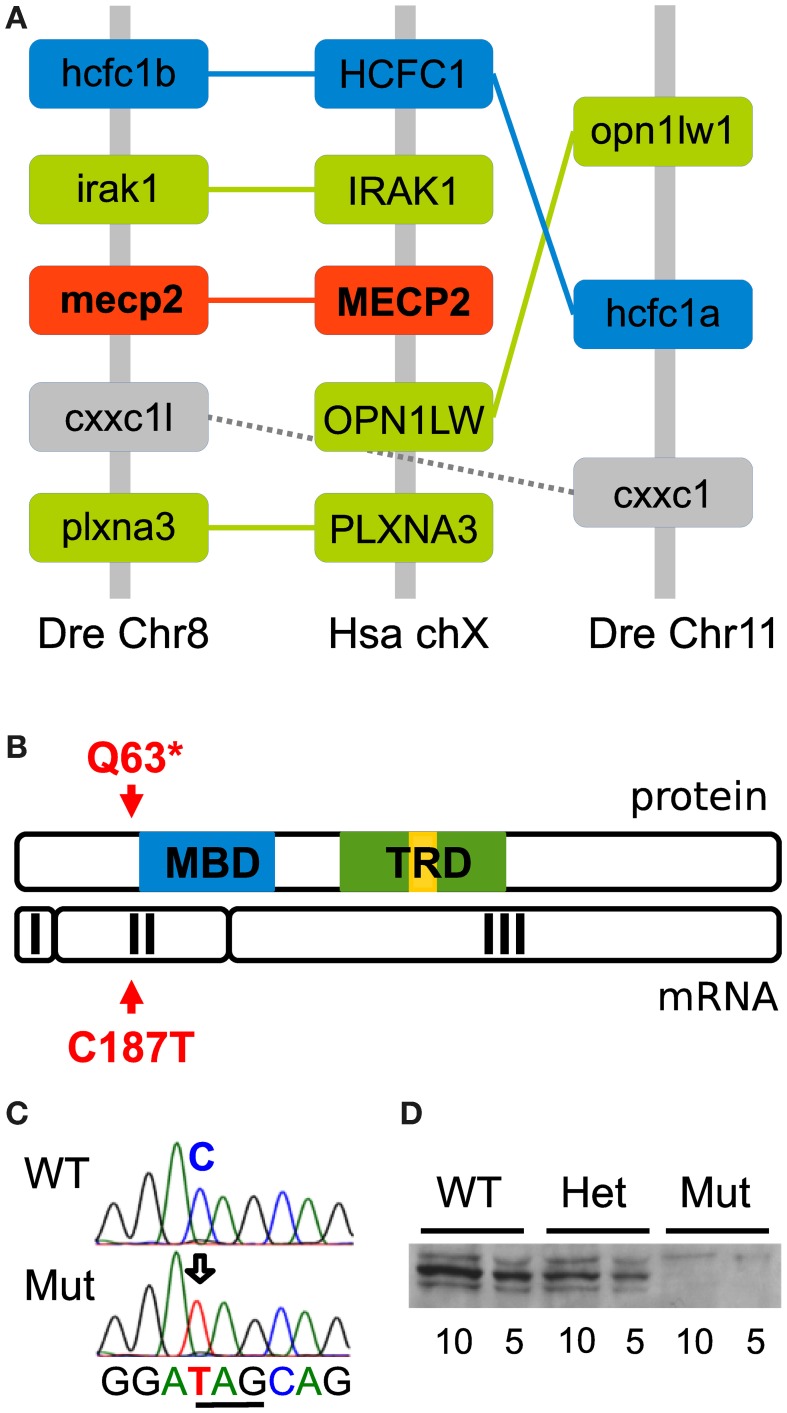
Conserved synteny and generation of the zebrafish mecp2^*Q*63*^ mutation. (A) Synteny analysis between zebrafish chromosome 8 and 11 and human chromosome X. The diagram shows regions of 2.07 Mb and 0.07 Mb, respectively, in zebrafish chromosome 8 and 11 and 0.5 Mb in human chromosome X. The paralog hcfc1 group and ortholog to HCFC1 is indicated in blue, others in green. In addition, the second indicated paralog group is depicted in gray and mecp2 in red. The structure of these genomic regions shows that mecp2 is unique in the zebrafish genome and an ortholog of the human MECP2. **(B)** TILLING of ENU-mutagenized fish sperm after screening of exon 2 has allowed for the recovery of a C187T transition, creating a non-sense mutation. This mutation is upstream of the MBD and TRD functionnal domains, causing putatively a null mutation. **(C)** Sequence of mecp2 PCR from genomic DNA confirmed the C to T transition and the creation of a stop codon (underlined). **(D)** Western blot analysis of wild-type, mecp^*Q*63*/+^ and mecp^*Q*63*/*Q*63*^ adult zebrafish brain tissue. Two quantities of proteins (10 and 5 μg of total lysate) were loaded for each genotype. A major band around 75 kD reveals the presence of mecp2 in wild-type and mecp^*Q*63*/+^, but not in mecp^*Q*63*/*Q*63*^ adult zebrafish brain (minor bands might represent alternative post-translational mecp2 forms).

Tetrapod vertebrates possess one copy of the *mecp2* gene located on the X chromosome. However, during evolution, teleost fish underwent an additional round of full genome duplication, leading to the possibility that in zebrafish two copies of the *mecp2* gene are present. Synteny analysis was therefore carried out to compare the genomic region of the human chromosome X flanking the MECP2 gene to the zebrafish genome. After reciprocal BLASTp searches with the sequences of the human proteins inferred from the genes flanking human MECP2, we found two syntenic regions in chromosome 8 and chromosome 11 (Figure 1A), in which zebrafish genes orthologous to the human genes flanking MECP2 were detected. Moreover, several paralog genes were present in these two zebrafish genomic regions, covering 2.07 megabases (Mb) in chromosome 8 and 0.07 Mb in chromosome 11 (only the cxxc1 and hcfc1 paralog group has been indicated for clarity) and flank the *mecp2* gene in chromosome 8. The comparison of the genomic organization of these two regions enabled us to conclude that the zebrafish genome has retained only one copy of the *mecp2* gene which is the ortholog of the human MECP2.

### Characterization of the *mecp2*^Q63*^ mutation

Screening of the two last exons of *mecp2* in ENU-mutagenized zebrafish sperm allowed us to recover by TILLING a C to T transition near the 5′ part of the *mecp2* coding sequence, at position 187, causing a non-sense mutation and putatively truncating the protein at position 63. The mutant allele would then encode a form of the protein lacking both the crucial methyl-CpG binding (MBD) and the transcription repressor (TRD) functional domains (Figure 1B) most probably engendering a full loss of function of mecp2. C to T transition in homozygote mutants was confirmed by sequencing *mecp2* PCR-amplified genomic DNA fragments, showing the creation of a non-sense amber codon in place of a glutamine codon in position 187/189 (Figure 1C). F1 generation was outcrossed in the AB background to remove potential mutations carried along with the *mecp*^*Q*63*^ allele, heterozygotes (*mecp*^*Q*63*/+^) were outcrossed for at least 3 generations before to be incrossed to produce homozygote mutants (*mecp*^*Q*63*/*Q*63*^). Surprisingly, the homozygote *mecp2* mutant fish appears to develop normally, with no obvious morphological defects, they reach adulthood and reproduce. As maternally expressed *mecp2* mRNA has been detected in large amounts (Coverdale et al., 2004), *mecp*^*Q*63*/*Q*63*^ fish were incrossed to remove any potential maternal contribution toward a milder phenotype. Similarly to the *mecp*^*Q*63*/*Q*63*^ from *mecp*^*Q*63*/+^ parents, the progeny of *mecp*^*Q*63*/*Q*63*^ fish develop apparently normally and are viable. Thus, maternal *mecp2* mRNA does not play a role in the moderation of the phenotype. However, albeit not quantified, in comparison to wild-type or *mecp*^*Q*463*/+^ fish, *mecp*^*Q*63*/*Q*63*^ fish appear to have a slightly shorter lifespan. This lighter-than-expected phenotype in comparison to the mecp2-null mouse models could be explained by the fact that the mutation does not produce a null allele. Therefore, to assess how the mutation affects the protein level in the homozygote *mecp2* mutant, 5 and 10 μ g of adult brain proteins from wild-type, *mecp*^*Q*63*/+^ and *mecp*^*Q*63*/*Q*63*^ fish were loaded on gels and subjected to Western blotting, with an antibody recognizing the C-terminus of the protein. In contrast to wild-type and heterozygote mutant fish, mecp2 protein could not be detected in *mecp*^*Q*63*/*Q*63*^ fish (Figure 1D), indicating that the C187T transition is very likely to produce a null allele.

### Early motor behavior

**Figure 2:**
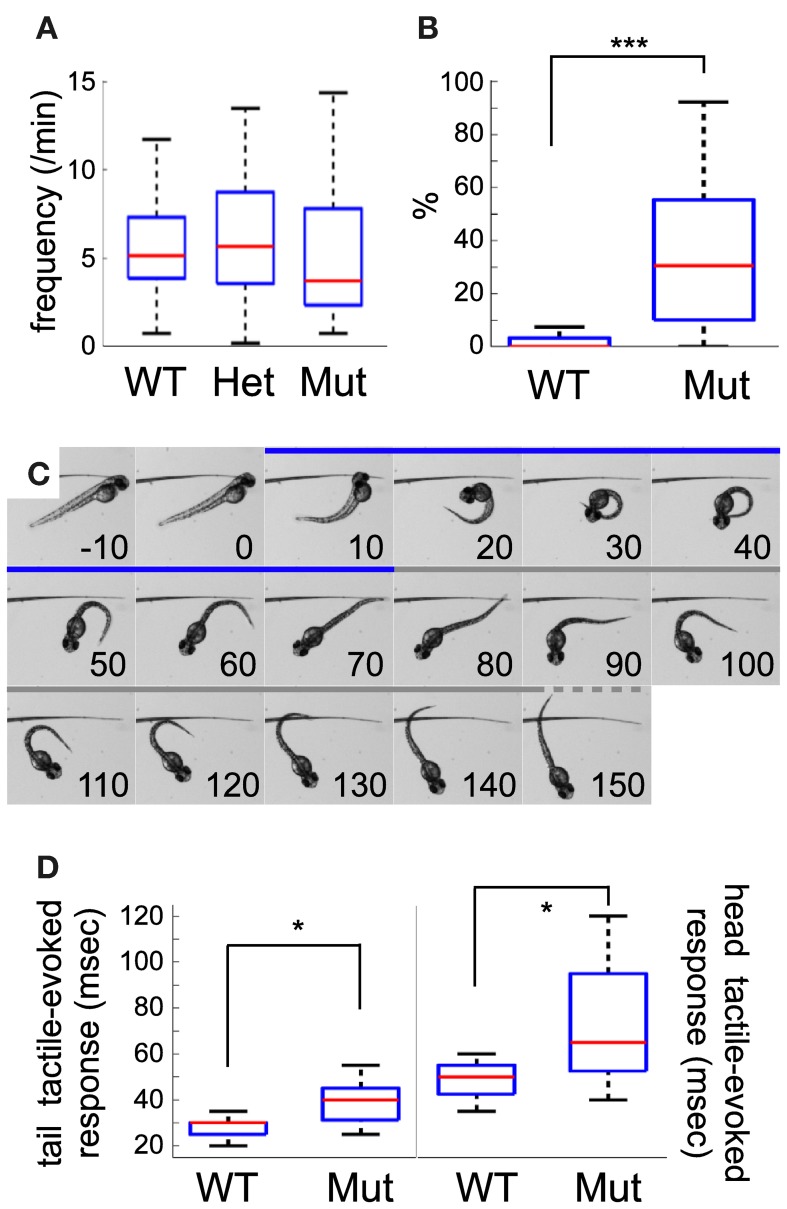
**Comparison between wild-type and mecp2-mutant embryo motor behavior. (A)** Frequency of coiling in wild-type, mecp^*Q*63*/+^ and mecp^*Q*63*/*Q*63*^ 25 hpf embryos. No statistical difference was detected between the three groups of embryos. **(B)** mecp^*Q*63*/*Q*63*^ 25 hpf embryos showed a significant larger number of multiple contractions per event than wild-type embryos. **(C)** Selected frames from high-speed video sequences showing a head-tactile-evoked response of a mecp^*Q*63*/*Q*63*^ 51 hpf embryo. The coiling and swim bout periods are depicted in blue and gray, respectively. After the stimulation, the embryo performs an escape-like response, moving away from the stimulus, through a classical C-bend flexion. Time, in msec, is indicated in each panel. **(D)** Kinematic quantification of tail and head tactile stimulation in wild-type and mecp^*Q*63*/*Q*63*^ 51 hpf embryos. Duration of the C-bend response to tail stimulation is shorter than head stimulation. A significant statistical difference between the two groups of embryos was observed for the duration of tail as well as head-evoked coilings. Significant statistical differences are indicated by ^*^ for *p* < 0.05 and ^***^ for *p* < 0.001.

Spontaneous contractions were monitored in 25 hpf dechorionated embryos at 21–22°C. Wild-type embryos mainly showed unique side coiling of the trunk separated by a period of rest of several seconds. However, in some occasions, the contraction events led to several successive coilings without noticeable delay between each movement. Isolated, as well as multiple contraction movements were considered as single spontaneous motor activity events. The spontaneous motor activity of wild-type, heterozygote, and homozygote *mecp2* mutant embryos was then quantified. In line with a previous report (Pietri et al., 2009), wild-type embryos presented a median frequency of coiling of 5.12 events/min (*n* = 71) at 25 hpf (Figure 2A), not significantly different from *mecp2*^Q63*/+^ or *mecp2*^Q63*/Q63*^ embryos, with 5.66 events/min (*n* = 33) and 3.70 events/min (*n* = 33), respectively. Interestingly, the *mecp2*^Q63*/Q63*^ embryos showed higher activity in comparison to the others, with a larger number of contractions per event in contrast to the wild-type and *mecp2*^Q63*/+^ embryos. During the recording period, 80.6% of the *mecp2*^Q63*/Q63*^ embryos exhibit at least one event with more than one contraction, in contrast to 24.3% of the other embryos. Overall, a significant median increase was observed in *mecp2*^Q63*/Q63*^ embryos, where 30.55% of the events were composed of multiple coilings, in comparison to wild-type embryos (0%, *p* < 10^−8^, Figure 2B).

To further study the early development of motor behavior of the *mecp2*^Q63*/Q63*^ fish, we characterized the escape response to tactile stimuli of 51 hpf embryos. In contrast to spontaneous activity or stimulus-evoked response in earlier embryos, where only the local circuit within the spinal cord is necessary and sufficient to produce motor behaviors (Pietri et al., 2009), escape-induced stimuli in 51 hpf embryos involves hindbrain structures, in particular the reticulospinal neurons (O'Malley et al., 1996; Liu and Fetcho, 1999). These cells notably receive inputs from the spinal sensory Rohon-Beard cells (Fetcho and Faber, 1988), and the multi-sensory recipient trigeminal ganglia afferents innervating the skin of the head (Kimmel et al., 1990). To test both pathways, we mechanically stimulated the trunk or the head of the embryos (see Figure 2C as an example of response to head tactile stimulation). Both types of stimulus produced an escape-like behavior, with embryos turning away from the stimulus in a characteristic C-bend followed by a burst of swim bouts. Figure 1C shows an example of a 51 hpf *mecp2*^Q63*/Q63*^ embryo where tactile stimulation of the head (0 ms) induced a C-bend (coiling period: 10–70 ms), and a swim bout (from 80 ms onward). In wild-type embryos, head and tail tactile stimulations produced behavioral responses with significantly different median C-bend durations [head (h, *n* = 11): 50 ms and tail (t, *n* = 20): 30 ms, *p* < 10^−5^, Figure 2D]. The same trend is observed between tail and head tactile stimulation responses in *mecp2*^Q63*/Q63*^ embryos [h (*n* = 16): 65 ms and t (*n* = 11): 40 ms, *p* = 0.0017], indicating that the principles governing both responses are conserved. Comparison of wild-type and *mecp2*^*Q*63*/*Q6*3*^ embryos for head or tail tactile stimulation revealed however, for both types of stimuli, a longer duration of the C-bend for the *mecp2*^Q63*/Q63*^ embryos (h: 65 ms vs. 50 ms, *p* = 0.0113; and t: 40 ms vs. 30 ms, *p* = 0.0151).

### Spontaneous swimming activity

**Figure 3:**
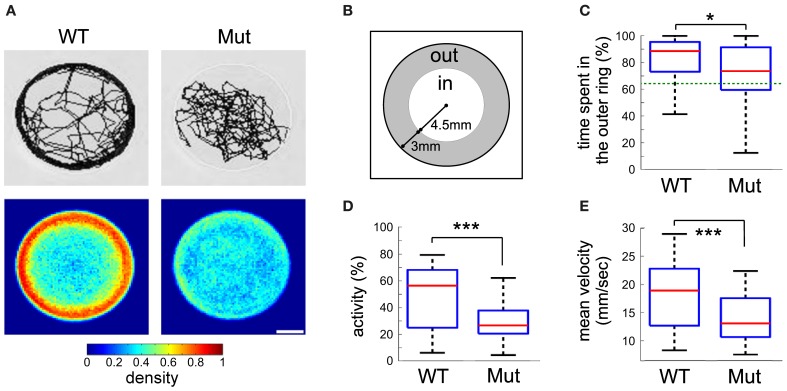
Kinematic analysis of free swimming wild-type and mecp^*Q*63*/*Q*63*^ 6 dpf larvae. (A) Example of traces for wild-type and mecp^*Q*63*/*Q*63*^ larvae, obtained from a recording session of 15 min (top), and density maps representing the proportion of larvae at each position in the well (bottom, *n* = 80 for both groups of larvae). **(B)** The arena-surface division into inner and outer regions as used for data analysis. **(C)** Quantification of the time spent in the inner and outer regions of **(B)**. Dotted green line is the random case. **(D)** Percentage of time when the larvae were active. **(E)** Mean velocity during activity. Significant statistical differences are indicated by ^*^ for *p* < 0.05 and ^***^ for *p* < 0.001.

**Figure 4:**
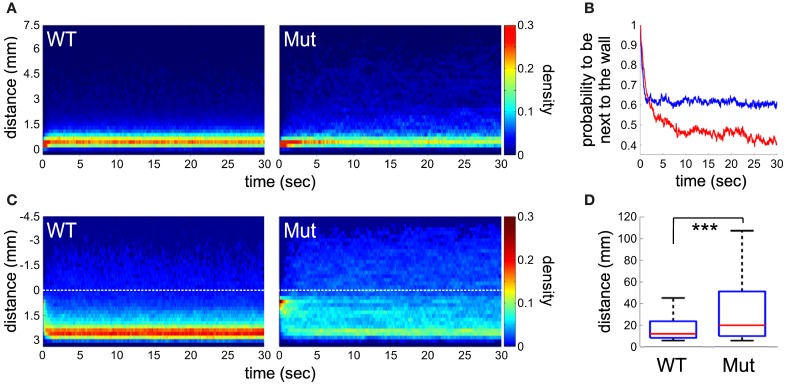
**Analysis of trajectories and thigmotaxis. (A)** Place preference of the larvae after they reached the edge of the wall (< 0.5 mm) during a period of 30 s. Density plot representing, for each time point, the proportion of larvae at a given distance from the well's edge (*n* = 80 for both groups of larvae). **(B)** The probability of larvae to remain next to the wall edge (< 1 mm) along time. Statistical difference is observed after 5 s (*p* < 0.001), between mecp^*Q*63*/*Q*63*^ (in red) and wild-type (in blue) larvae. **(C)** Trajectory analysis of the larvae after crossing from the inner to outer region (color coded as in **A**). The dotted lines indicate the boundary between the two regions. **(D)** The length of the paths from the center (a disc representing 5% of the total area) to the well's edge. Significant statistical differences are indicated by ^***^ for *p* < 0.001.

Differences between wild-type and *mecp2* mutant fish were also assessed by monitoring their spontaneous motor behavior at 6 dpf. We found that the thigmotactic responses of *mecp2*^Q63*/Q63*^ larvae were different from their wild-type counterparts (Figures 3, 4). Wild-type animals preferred to swim along the walls of the arena, while *mecp2*^Q63*/Q63*^ larvae showed much less preference for the borders of the well, covering homogeneously most of its surface (Figure 3A for a single animal and for an average of *n* = 80, for both groups of larvae). We performed a quantitative analysis of the preferred swimming regions according to an inner region of radius 4.5 mm and an outer ring to the limit of the circular arena of radius 7.5 mm (Figure 3B). Wild-type animals spent 88.5 % of the time in the outer ring while *mecp2*^Q63*/Q63*^ larvae 73 % (*p* = 0.001, Figure 3C), closer to the random case of 64% (dashed green line, Figure 3C). To discriminate whether this difference had a motor origin or was due to the different interactions with the wall, we analyzed their trajectories to and from the walls. In addition, we found that the distribution of the resting times of wild-type and *mecp2*^Q63*/Q63*^ larvae could be fitted to the same power law *Y* = aX^−k^, by adjusting the constant *k* accordingly. In comparison to wild-type, *mecp2*^Q63*/Q63*^ larvae showed a significantly lower median *k* constant (0.0525 vs. 0.2399 for wild-type larvae, *p* = 0.0007). In a reciprocal way, active time bouts had a larger *k*-value in *mecp2*^Q63*/Q63*^ larvae (2.79 vs. 1.92 for wild-type larvae, *p* = 0.0051). These altered values of *k* are coherent with the lower total motor activity (26.4 % vs. 56.0 % *p* < 10^−6^, Figure 3D), and lower bout velocities (13.0 mm/sec vs 18.9 mm/sec, *p* < 10^−7^, Figure 3E) for *mecp2*^Q63*/Q63*^ in comparison to wild-type larvae. These results would then predict that *mecp2*^Q63*/Q63*^ larvae already at the outer ring would stay longer at this location, but we observed the opposite (Figures 4A,B). Analysis of the portion of the trajectories starting when the larvae reached the wall edge showed that most *mecp2*^Q63*/Q63*^ larvae had a lower probability to stay in this region than their wild-type counterparts (Figures 4A,B). Interestingly, for the first 2 s, *mecp2*^Q63*/Q63*^ larvae had a higher probability of staying at the wall, consistent with the lower activity and velocities, but this quickly decreased afterwards to values below those of wild-type larvae, with significant differences after 5 s, reaching *p* < 10^−10^ at 30 s. We also analyzed the portion of trajectories moving from the center toward the wall (using the inner/outer separation as *t* = 0) and found that *mecp2*^Q63*/Q63*^ larvae showed a significant lower probability in reaching the wall (after 30 s: *p* < 10^−9^, Figure 4C). To test if the latter is a consequence of the lower activity and velocity, we computed the length of the path from the center (within an area of less than 5% of the well) to the wall; a parameter completely independent of activity and velocity. *mecp2*^Q63*/Q63*^ larvae showed significantly longer paths than wild-type larvae (19.9 mm vs 12 mm, *p* < 10^−13^, Figure 4D). Overall, these results suggest that *mecp2*^Q63*/Q63*^ larvae have impaired locomotion and decreased thigmotaxis.

## Discussion

In this study, we introduce the first mecp2-null RTT zebrafish model. In comparison to other mecp2-deficient animal models, this mutant shows a weaker phenotype as they are viable and fertile with a seemingly shorter lifespan. However, it shows clear behavioral impairments.

### Knock-out of mecp2 in zebrafish produces an unexpectedly mild phenotype

In this model, suppression of the mecp2 functions was achieved by TILLING selection of a non-sense mutation upstream of the structural MBD domain. The position of the mutation within the gene predicted to be a null mutation. This prediction was supported by protein expression analysis that confirmed the complete loss of mecp2 in *mecp2*^Q63*/Q63*^ zebrafish. In mouse, null mutations of MeCP2 induce phenotypes reminiscent of RTT (Chen et al., 2001; Guy et al., 2001). In particular, the lifespan of mutant mice is drastically reduced to 6 to 8 weeks after birth. To our surprise *mecp2*^Q63*/Q63*^ zebrafish are viable and reproduce normally (in presence or absence of unaltered mecp2 maternal contribution). We noticed however, a tendency of this mutant fish to have a relatively shorter lifespan than wild-type zebrafish. This modest phenotype with respect to mice may indicate functional compensatory mechanisms, for instance, by the presence of a *mecp2* paralog gene. Synteny mapping, comparing human and zebrafish *mecp2* loci and extensive genome database mining of zebrafish and other teleost fish [in which the whole-genome duplication occurred before the radiation of these species (not shown; Postlethwait et al., 2004)] excludes the existence of *mecp2* paralog groups in the various fish species tested, indicating that the loss of the duplicated *mecp2* gene occurred probably early after the whole teleost genome duplication, and so in zebrafish. Partial functional compensation may also exist through the action of close MeCP2 protein family members, such as MBD2 that, like MeCP2 bears MBD and TRD domains. MBD2 is able to bind to methylated CpG dinucleotides and modulates the level of gene expression (Ng et al., 1999). This hypothesis has been raised to point out the lack of phenotype in null-MeCP2 mutant mouse in non-neural tissues, where MeCP2 is also expressed (Guy et al., 2001); this possibility has however not been thoroughly tested yet. In addition, due to the duplication of many genes in the zebrafish genome, compensatory mechanisms can take place at the level of the *mecp2* target genes. For instance, MeCP2-deficient mice present various alterations of NMDA receptor expressions that are region and age specific (Blue et al., 2011). In zebrafish, all five NMDA receptors present in mammals are duplicated (Cox et al., 2005), raising the possibility that *mecp2*-induced alterations of the expression of one of the genes in a paralog group is compensated by expression of the second one. Moreover, these receptors are heteromeric, and if compensation within a paralog group cannot be achieved, alternative forms of the receptors could, at least partially, be used. Finally, the difference in the phenotypes between mouse and zebrafish might be explained by the far greater regenerative abilities of the zebrafish brain than those of the mammalian ones. In contrast to mammals, zebrafish neurogenesis occurs throughout life in many brain regions (Zupanc, 2008). While new-born neurons integrate into already established networks, “old” neurons are eliminated by apoptosis. The late onset of the neural phenotype of the MeCP2-deficient mouse (Chen et al., 2001; Guy et al., 2001) and postnatal neural restoration of MeCP2 expression in MeCP2-null mouse were shown to be sufficient to largely rescue the RTT phenotypes (Guy et al., 2007), thus, suggesting that alteration of MeCP2 functions perturbs the maintenance of the nervous system rather than its initial development. It is conceivable that the constant addition of new neurons in zebrafish neural circuits could prevent the appearance of the lethal phenotype seen in MeCP2-null mouse models. These various compensatory mechanisms that are for some of them uniquely present in zebrafish, may explain its high resilience to the loss of *mecp2*. On the other hand, the ENU-mutagenesis used in the TILLING procedure produces a large number of mutations. Some may be carried along with the mutation of interest, even after several generations. We thus cannot completely exclude the presence of a second genetically-linked mutation to *mecp2*^Q63*^. Our mecp2-null model presents a milder-than-expected phenotype. Thus, if indeed zebrafish and mammals mecp2 functions are conserved, a second genetic alteration would have to be a suppressor of the effect of the *mecp2*^Q63*^ mutation. The probability of such mutation in this context is very low. In-depth sequencing or s*ingle nucleotide polymorphisms* analysis of the mecp2 region could be used to rule out this concern.

### Spontaneous and evoked early motor alterations suggest an excitation/inhibition unbalance

By 24 hpf, spontaneous motor activity seems to be triggered by an intraspinal premotor circuit (Saint-Amant and Drapeau, 1998; Pietri et al., 2009). At this developmental stage, the excitatory components rely on electrical coupling via gap junctions, reinforced by nascent glutamatergic synapses (Saint-Amant and Drapeau, 1998; Pietri et al., 2009). On the other hand, inhibition appears to be driven only by glycinergic inputs, as evidence of GABAergic participation in the early spontaneous activity could not be detected (Saint-Amant and Drapeau, 2000, 2001). The similarity in the frequency of spontaneous muscular contractions between wild-type and mutant mecp2 embryos suggests that excitation in the spinal cord may remain intact in *mecp2*^Q63*/Q63*^ embryos, while the sustained spontaneous motor activity events in these embryos would argue for a decrease in inhibition efficacy, likely through perturbation of the glycinergic system. A less parsimonious hypothesis would be that both excitation and inhibition are depressed in 25 hpf *mecp2*^Q63*/Q63*^ embryos, but with a stronger alteration of the glycinergic inhibitory component. This hypothesis is supported by the observed tendency for a lower rate of spontaneous activity events in 25 hpf *mecp2*^Q63*/Q63*^ embryos. In contradiction with these hypothesis, one study on the medulla of MeCP2-null mouse at postnatal day 7 has shown that glycinergic transmission is unaltered, while GABAergic transmission is strongly depressed (Medrihan et al., 2008). However, modulation of the defects of particular neurotransmitters appears to be region and age specific (Blue et al., 2011). It is therefore plausible that alteration of glycinergic transmission is either transient or only effective in the spinal cord of *mecp2*^Q63*/Q63*^ fish.

One of the symptoms observed in autistic patients, including RTT patients, is their hyper-responsiveness to sensory stimuli (Belmonte et al., 2004). To investigate sensorimotor behavior in zebrafish early development, light mechanical stimuli were thus applied on the trunk or the head of the embryos to induce a startle/escape behavior. By 2 dpf, head and tail sensory stimuli are integrated through hindbrain reticulospinal neurons to produce a motor response. These sets of neurons receive inputs from the sensory trigeminal and Rohon-Beard neurons innervating respectively the head and body skin (Kimmel et al., 1990; Kuwada et al., 1990). While they appear similar at 21 hpf (Saint-Amant and Drapeau, 1998), responses to head and tail tactile stimulations showed different kinematics in 4–5 dpf larvae (Liu and Fetcho, 1999; Liu et al., 2012), reflecting the involvement of different reticulospinal neurons in mediating the escape response (Gahtan et al., 2002). In 51 hpf embryos, tail tactile stimulations induced coiling responses of shorter durations than those evoked by head stimulations, similar to what was found at 5 dpf, and thus suggesting that t different sets of reticulospinal neurons are already required at an early stage of development. *mecp2*^Q63*/Q63*^ embryos responded to tactile stimulations showing a similar difference between the duration of head and tail-induced responses seen in wild-type embryos. This suggests that the structure of the network necessary for both sensory-evoked motor responses is not altered. However, careful examinations of the mouse models, previously believed to be asymptomatic in their early development, have uncovered alterations of their sensory reflexes and sensory-motor gating development (Picker et al., 2006; Santos et al., 2007). Similarly, at 51 hpf, *mecp2*^Q63*/Q63*^ embryos showed alterations of sensory-evoked motor responses characterized by a lengthening of the coiling duration. Both trigeminal and Rohon-Beard sensory neurons implicated in the transmission of the mechanical stimulation as well as the Mauthner neurons are glutamatergic neurons (Higashijima et al., 2004). At this age, glycinergic neurotransmition appears to remain the main inhibitory system in the spinal cord (Saint-Amant, 2010). Similar alteration of the coiling responses of both sensory stimulations in *mecp2*^Q63*/Q63*^ embryos may imply that only the premotor network (downstream of the reticulospinal neurons) is affected and like in earlier stage embryos, the perturbation of the glycinergic system is responsible for the observed phenotype. Alternatively, glutamatergic neurotransmission may be altered in a similar way in both types of sensory neurons. This hypothesis can not be ruled out, as our experiments did not allow for the quantification of the delay of the response to a tactile stimulation, which would be indicative of such perturbation. It is therefore possible that defects in glutamatergic excitation are combined with alterations of the inhibitory neurotransmission, as seen in the mouse model of RTT (Chao et al., 2007, 2010; Medrihan et al., 2008; Blue et al., 2011).

### Mecp2-mutant larvae showed reduction in activity and a decrease in anxiety-like behavior

Patients with RTT suffer from pronounced alterations of their motor systems. These defects have been phenocopied in RTT mouse models. We therefore tested our zebrafish model for motor dysfunctions in 6 dpf larvae in the open-field paradigm. As seen in mouse models, alteration of *mecp2* functions in zebrafish larvae induced a sharp decrease of the activity level, notably characterized by an increase of the resting times between swimming bouts. Interestingly, both resting times in wild-type and *mecp2*^Q63*/Q63*^ larvae followed the same power-law distribution. The fact that a power law can describe the kinematics of both *mecp2*^Q63*/Q63*^ and wild-type larvae suggests that the mutation modulates wild-type neural networks rather than generating major muscular or/and morphological abnormalities that could indirectly affect motor behavior. (Harnos et al., 2000; Faure et al., 2003). In mecp2-null mouse models, the different aminergic systems have been shown to be specifically defective (Panayotis et al., 2011). In particular, defects in the dopaminergic system have been shown to be responsible for the movement abnormalities (Samaco et al., 2009; Gantz et al., 2011). In zebrafish, dopaminergic systems regulate the development of locomotor circuits, putatively by regulating synaptogenesis or influencing other neuromodulatory pathways (Thirumalai and Cline, 2008). It is thus possible that suppression of *mecp2* functions in zebrafish larvae induces a perturbation of neuromodulation of the motor network development that would then underlie the motor phenotype.

The dopaminergic, among other neuromodulatory systems, is also implicated in the control of anxiety-related behaviors (Wood and Toth, 2001). Thus, we tested for thigmotaxis in 6 dpf larvae, a behavioral test commonly used to evaluate the level of anxiety in humans and other animals. In zebrafish, this test has been particularly used in adults (Peitsaro et al., 2003; Anichtchik et al., 2004), but recently validated in larvae as young as 5 dpf (Colwill and Creton, 2011; Schnorr et al., 2012). In larvae, as well as in adults, thigmotaxis has been interpreted as a defensive strategy to avoid detection by predators and is reduced in familiar environments (Colwill and Creton, 2011) or after exposure to anxiolytic drugs (Schnorr et al., 2012). Studies of anxiety in mouse models of RTT have brought so far inconsistent results on the presence of elevated or reduced anxiety-like behaviors. The complexity of this behavior involving several brain structures, neurotransmitter systems and hormonal neuromodulation makes the interpretation of the various tests difficult and may thus explain such discrepancy. However, place preference analysis in an open-field arena, which would be the equivalent of wells for zebrafish, have shown an increase in anxiety levels in 3 and 5 month old MeCP2^308^ mice (Shahbazian et al., 2002b) and in 8-week old mecp2-null mice (Stearns et al., 2007). This is in contradiction with our results showing that *mecp2*^Q63*/Q63*^ larvae spent less time close to the well's edge than wild-type larvae. However, motor dysfunction present in the mutant larvae may lead to false interpretation of the place-preference results. Thus, a subset of trajectories were chosen to test for attraction toward edges and maintenance of edge preference during the active phases of swimming. *mecp2*^Q63*/Q63*^ larvae showed a clear reduction in both parameters, strongly suggesting a decreased level of thigmotaxis in comparison to wild-type age-matched larvae. Moreover, the mutant larvae covered more distance in the inner region of the well than their wild-type counterparts in their periods of activity, a variable that has also been shown to be indicative of the level of anxiety in mice in the open-field paradigm (Paylor et al., 1998). Overall, these results suggest that *mecp*^*Q*63*/*Q*63*^ larvae are less anxious than their wild-type counterparts.

Several autistic features have also been explained as a possible dysfunction of the reward system (Scott-Van Zeeland et al., 2010; Dichter et al., 2012). Interestingly, the dopaminergic system is also a core component of the reward circuits (Dichter et al., 2012). The decrease in locomotor activity of *mecp*^*Q*63*/*Q*63*^ larvae could thus be explained as a reduction of interest in environment exploration. The decreased thigmotaxis could be the result of avoiding tactile stimulation from the wall, as expected from hyper-responsive autistic patients (Belmonte et al., 2004).

## Conclusion

While rodents have been the historical model of choice to study the genetic alterations present in neurodevelopmental and neurodegenerative human diseases, zebrafish is currently becoming an important complementary model for translational neuroscience research, as it is the only suitable vertebrate model for high throughput drug screening. Analysis of *mecp2*-deficient zebrafish during early development has uncovered motor defects compatible with motor phenotypes observed in MeCP2-null mouse models and RTT patients. The zebrafish RTT model, in contrast to other vertebrate ones, will enable monitoring brain dynamics, neural morphology and behaviors, through the entire normal lifespan of the organism. Furthermore, the non-lethality of this model will enable the study of the natural genetic compensatory mechanisms. Deciphering these mechanisms would potentially lead to alternative directions in the understanding of the pathophysiology of RTT and open the way for the discovery of novel treatments.

### Conflict of interest statement

The authors declare that the research was conducted in the absence of any commercial or financial relationships that could be construed as a potential conflict of interest.

## References

[B1] Amir R. E. Van den Veyver I. B. Wan M. Tran C. Q. Francke U. Zoghbi H. Y. (1999). Rett syndrome is caused by mutations in X-linked MeCP2, encoding methyl-CpG-binding protein 2. Nat. Genet. 23, 185–188 10.1038/13810 10508514

[B2] Anichtchik O. V. Kaslin J. Peitsaro N. Scheinin M. Panula P. (2004). Neurochemical and behavioural changes in zebrafish *Danio rerio* after systemic administration of 6-hydroxydopamine and 1-methyl-4-phenyl-1,2,3,6-tetrahydropyridine. J. Neurochem. 88, 443–453 10.1111/j.1471-4159.2004.02190.x 14690532

[B3] Ballas N. Lioy D. T. Grunseich C. Mandel G. (2009). Non-cell autonomous influence of MeCP2-deficient glia on neuronal dendritic morphology. Nat. Neurosci. 12, 311–317 10.1038/nn.2275 19234456 PMC3134296

[B4] Beattie C. E. Carrel T. L. McWhorter M. L. (2007). Fishing for a mechanism: using zebrafish to understand spinal muscular atrophy. J. Child Neurol. 22, 995–1003 10.1177/0883073807305671 17761655

[B5] Belmonte M. K. Cook E. H. Jr. Anderson G. M. Rubenstein J. L. Greenough W. T. Beckel-Mitchener A. (2004). Autism as a disorder of neural information processing: directions for research and targets for therapy. Mol. Psychiatry 9, 646–663 15037868 10.1038/sj.mp.4001499

[B6] Blue M. E. Kaufmann W. E. Bressler J. Eyring C. O'Driscoll C. Naidu S. (2011). Temporal and regional alterations in NMDA receptor expression in MeCP2-null mice. Anat. Rec. (Hoboken) 294, 1624–1634 10.1002/ar.21380 21901842 PMC4122218

[B7] Brennan C. H. (2011). Zebrafish behavioural assays of translational relevance for the study of psychiatric disease. Rev. Neurosci. 22, 37–48 10.1515/rns.2011.006 21615260

[B8] Chahrour M. Jung S. Y. Shaw C. Zhou X. Wong S. T. C. Qin J. (2008). MeCP2, a key contributor to neurological disease, activates and represses transcription. Science 320, 1224–1229 10.1126/science.1153252 18511691 PMC2443785

[B9] Chao H. Zoghbi H. Y. Rosenmund C. (2007). MeCP2 controls excitatory synaptic strength by regulating glutamatergic synapse number. Neuron 56, 58–65 10.1016/j.neuron.2007.08.018 17920015 PMC2198899

[B10] Chao H. Chen H. Samaco R. C. Xue M. Chahrour M. Yoo J. (2010). Dysfunction in GABA signaling mediates autism-like stereotypies and Rett syndrome phenotypes. Nature 468, 263–269 10.1038/nature09582 21068835 PMC3057962

[B11] Chen R. Z. Akbarian S. Tudor M. Jaenisch R. (2001). Deficiency of methyl-CpC binding protein-2 in CNS neurons results in a Rett-like phenotype in mice. Nat. Genet. 27, 327–331 10.1038/85906 11242118

[B12] Colwill R. M. Creton R. (2011) Locomotor behaviors in zebrafish (*Danio rerio*) larvae. Behav. Processes. 86, 222–229 10.1016/j.beproc.2010.12.003 21147203 PMC3063417

[B13] Coverdale L. E. Martyniuk C. J. Trudeau V. L. Martin C. C. (2004). Differential expression of the methyl-cytosine binding protein 2 gene in embryonic and adult brain of zebrafish. Brain Res. Dev. Brain Res. 153, 281–287 10.1016/j.devbrainres.2004.08.009 15527897

[B14] Cox J. A. Kucenas S. Voigt M. M. (2005) Molecular characterization and embryonic expression of the family of N-methyl-D-aspartate receptor subunit genes in the zebrafish. Dev. Dyn. 234, 756–766 10.1002/dvdy.20532 16123982

[B15] Curado S. Anderson R. M. Jungblut B. Mumm J. Schroeter E. Stainier D. Y. R. (2007). Conditional targeted cell ablation in zebrafish: a new tool for regeneration studies. Dev. Dyn. 236, 1025–1035 10.1002/dvdy.21100 17326133

[B16] Dani V. S. Chang Q. Maffei A. Turrigiano G. G. Jaenisch R. Nelson S. B. (2005). Reduced cortical activity due to a shift in the balance between excitation and inhibition in a mouse model of Rett syndrome. Proc. Natl. Acad. Sci. U.S.A. 102, 12560–12565 10.1073/pnas.0506071102 16116096 PMC1194957

[B17] Dichter G. S. Damiano C. A. Allen J. A. (2012). Reward circuitry dysfunction in psychiatric and neurodevelopmental disorders and genetic syndromes: animal models and clinical findings. J. Neurodev. Disord. 4:19 10.1186/1866-1955-4-19 22958744 PMC3464940

[B18] Draper B. W. McCallum C. M. Stout J. L. Slade A. J. Moens C. B. (2004). A high-throughput method for identifying N-ethyl-N-nitrosourea (ENU)-induced point mutations in zebrafish. Methods Cell Biol. 77, 91–112 10.1016/S0091-679X(04)77005-3 15602907

[B19] Faure P. Neumeister H. Faber D. S. Korn H. (2003). Symbolic analysis of swimming trajectories reveals scale invariance and provides a model for fish locomotion. Fractals 11, 233–243 10.1142/S0218348X03002166

[B19a] Fetcho J. R. Faber D. S. (1988). Identification of motoneurons and interneurons in the spinal network for escapes initiated by the mauthner cell in goldfish. J. Neurosci. 8, 4192–4213 3183720 10.1523/JNEUROSCI.08-11-04192.1988 PMC6569477

[B20] Gahtan E. Sankrithi N. Campos J. B. O'Malley D. M. (2002). Evidence for a widespread brain stem escape network in larval zebrafish. J. Neurophysiol. 87, 608–614 11784774 10.1152/jn.00596.2001

[B21] Gantz S. C. Ford C. P. Neve K. A. Williams J. T. (2011). Loss of MeCP2 in substantia nigra dopamine neurons compromises the nigrostriatal pathway. J. Neurosci. 31, 12629–12637 10.1523/JNEUROSCI.0684-11.2011 21880923 PMC3201707

[B22] Guy J. Gan J. Selfridge J. Cobb S. Bird A. (2007). Reversal of neurological defects in a mouse model of Rett syndrome. Science 315, 1143–1147 10.1126/science.1138389 17289941 PMC7610836

[B23] Guy J. Hendrich B. Holmes M. Martin J. E. Bird A. (2001). A mouse MeCP2-null mutation causes neurological symptoms that mimic Rett syndrome. Nat. Genet. 27, 322–326 10.1038/85899 11242117

[B24] Harnos A. Horvath G. Lawrence A. B. Vattay G. (2000). Scaling and intermittency in animal behavior. Physica A 286, 312–320 10.1016/S0378-4371(00)00332-023516567

[B25] Higashijima S. Mandel G. Fetcho J. (2004). Distribution of prospective glutamatergic, glycinergic, and GABAergic neurons in embryonic and larval zebrafish. J. Comp. Neurol. 480, 1–18 10.1002/cne.20278 15515020

[B26] Huang P. Xiao A. Zhou M. Zhu Z. Lin S. Zhang B. (2011). Heritable gene targeting in zebrafish using customized TALENS. Nat. Biotechnol. 29, 699–700 10.1038/nbt.1939 21822242

[B27] Kabashi E. Brustein E. Champagne N. Drapeau P. (2011). Zebrafish models for the functional genomics of neurogenetic disorders. Biochim. Biophys. Acta 1812, 335–345 10.1016/j.bbadis.2010.09.011 20887784

[B28] Kimmel C. B. Hatta K. Metcalfe W. K. (1990). Early axonal contacts during development of an identified dendrite in the brain of the zebrafish. Neuron 4, 535–545 10.1016/0896-6273(90)90111-R 2322459

[B29] Kron M. Howell C. J. Adams I. T. Ransbottom M. Christian D. Ogier M. (2012). Brain activity mapping in MeCP2 mutant mice reveals functional deficits in forebrain circuits, including key nodes in the default mode network, that are reversed with ketamine treatment. J. Neurosci. 32, 13860–13872 10.1523/JNEUROSCI.2159-12.2012 23035095 PMC3500840

[B30] Kuwada J. Y. Bernhardt R. R. Nguyen N. (1990). Development of spinal neurons and tracts in the zebrafish embryo. J. Comp. Neurol. 302, 617–628 10.1002/cne.903020316 2262604

[B31] Lewis J. D. Meehan R. R. Henzel W. J. Maurer-Fogy I. Jeppesen P. Klein F. (1992). Purification, sequence, and cellular localization of a novel chromosomal protein that binds to methylated DNA. Cell 69, 905–914 10.1016/0092-8674(92)90610-O 1606614

[B32] Lioy D. T. Garg S. K. Monaghan C. E. Raber J. Foust K. D. Kaspar B. K. (2011). A role for glia in the progression of Rett's syndrome. Nature 475, 497–500 10.1038/nature10214 21716289 PMC3268776

[B33] Liu K. S. Fetcho J. R. (1999). Laser ablations reveal functional relationships of segmental hindbrain neurons in zebrafish. Neuron 23, 325–335 10.1016/S0896-6273(00)80783-7 10399938

[B34] Liu Y. Bailey I. Hale M. E. (2012). Alternative startle motor patterns and behaviors in the larval zebrafish (*Danio rerio*). J. Comp. Physiol. A Neuroethol. Sens. Neural. Behav. Physiol. 198, 11–24 10.1007/s00359-011-0682-1 21983742

[B36] Matsuishi T. Yamashita Y. Takahashi T. Nagamitsu S. (2011). Rett syndrome: the state of clinical and basic research, and future perspectives. Brain Dev. 33, 627–631 10.1016/j.braindev.2010.12.007 21232889

[B37] Medrihan L. Tantalaki E. Aramuni G. Sargsyan V. Dudanova I. Missler M. (2008). Early defects of GABAergic synapses in the brain stem of a MeCP2 mouse model of Rett syndrome. J. Neurophysiol. 99, 112–121 10.1152/jn.00826.2007 18032561

[B38] Moretti P. Bouwknecht J. A. Teague R. Paylor R. Zoghbi H. Y. (2005). Abnormalities of social interactions and home-cage behavior in a mouse model of Rett syndrome. Hum. Mol. Genet. 14, 205–220 10.1093/hmg/ddi016 15548546

[B39] Moretti P. Levenson J. M. Battaglia F. Atkinson R. Teague R. Antalffy B. (2006). Learning and memory and synaptic plasticity are impaired in a mouse model of Rett syndrome. J. Neurosci. 26, 319–327 10.1523/JNEUROSCI.2623-05.2006 16399702 PMC6674314

[B40] Morris J. A. (2009). Zebrafish: a model system to examine the neurodevelopmental basis of schizophrenia. Prog. Brain Res. 179, 97–106 10.1016/S0079-6123(09)17911-6 20302822

[B41] Ng H. H. Zhang Y. Hendrich B. Johnson C. A. Turner B. M. Erdjument-Bromage H. (1999). MBD2 is a transcriptional repressor belonging to the MeCP1 histone deacetylase complex. Nat. Genet. 23, 58–61 10.1038/12659 10471499

[B41a] O'Malley D. M. Kao Y.-H Fetcho J. R. (1996). Imaging the functional organization of zebrafish hindbrain segments during escape behaviors. Neuron 17, 1145–1155 10.1016/S0896-6273(00)80246-9 8982162

[B42] Panayotis N. Ghata A. Villard L. Roux J. C. (2011). Biogenic amines and their metabolites are differentially affected in the MeCP2-deficient mouse brain. BMC Neurosci. 12:47 10.1186/1471-2202-12-47 21609470 PMC3112112

[B43] Panula P. Sallinen V. Sundvik M. Kolehmainen J. Torkko V. Tiittula A. (2006). Modulatory neurotransmitter systems and behavior: towards zebrafish models of neurodegenerative diseases. Zebrafish 3, 235–247 10.1089/zeb.2006.3.235 18248264

[B44] Paylor R. Nguyen M. Crawley J. N. Patrick J. Beaudet A. Orr-Urtreger A. (1998). Alpha7 nicotinic receptor subunits are not necessary for hippocampal-dependent learning or sensorimotor gating: a behavioral characterization of Acra7-deficient mice. Learn Mem. 5, 302–316 10454356 PMC311270

[B45] Picker J. D. Yang R. Ricceri L. Berger-Sweeney J. (2006). An altered neonatal behavioral phenotype in mecp2 mutant mice. Neuroreport 17, 541–544 10.1097/01.wnr.0000208995.38695.2f 16543822

[B46] Pietri T. Manalo E. Ryan J. Saint-Amant L. Washbourne P. (2009). Glutamate drives the touch response through a rostral loop in the spinal cord of zebrafish embryos. Dev. Neurobiol. 69, 780–795 10.1002/dneu.20741 19634126 PMC2771646

[B47] Peitsaro N. Kaslin J. Anichtchik O. V. Panula P. (2003). Modulation of the histaminergic system and behaviour by alpha-fluoromethylhistidine in zebrafish. J. Neurochem. 86, 432–441 10.1046/j.1471-4159.2003.01850.x 12871584

[B47a] Postlethwait J. Amores A. Cresko W. Singer A. Yan Y. L. (2004). Subfunction partitioning, the teleost radiation and the annotation of the human genome. Trends Genet. 20, 481–490 10.1016/j.tig.2004.08.001 15363902

[B48] Saint-Amant L. (2010). Development of motor rhythms in zebrafish embryos.. Prog. Brain Res. 187, 47–61 10.1016/B978-0-444-53613-6.00004-6 21111200

[B49] Saint-Amant L. Drapeau P. (1998). Time course of the development of motor behaviors in the zebrafish embryo. J. Neurobiol. 37, 622–632 9858263 10.1002/(sici)1097-4695(199812)37:4<622::aid-neu10>3.0.co;2-s

[B50] Saint-Amant L. Drapeau P. (2000). Motoneuron activity patterns related to the earliest behavior of the zebrafish embryo. J. Neurosci. 20, 3964–3972 10818131 10.1523/JNEUROSCI.20-11-03964.2000 PMC6772631

[B51] Saint-Amant L. Drapeau P. (2001). Synchronization of an embryonic network of identified spinal interneurons solely by electrical coupling. Neuron 31, 1035–1046 10.1016/S0896-6273(01)00416-0 11580902

[B52] Samaco R. C. Mandel-Brehm C. Chao H. T. Ward C. S. Fyffe-Maricich S. L. Ren J. (2009). Loss of MeCP2 in aminergic neurons causes cell-autonomous defects in neurotransmitter synthesis and specific behavioral abnormalities. Proc. Natl. Acad. Sci. U.S.A. 106, 21966–21971 10.1073/pnas.0912257106 20007372 PMC2799790

[B52a] Santos M. Silva-Fernandes A. Oliveira P. Sousa N. Maciel P. (2007). Evidence for abnormal early development in a mouse model of Rett syndrome. Genes Brain Behav. 6, 277–286 10.1111/j.1601-183X.2006.00258.x 16848781

[B53] Scott-Van Zeeland A. A. Dapretto M. Ghahremani D. G. Poldrack R. A. Bookheimer S. Y. (2010). Reward processing in autism. Autism Res. 3, 53–67 20437601 10.1002/aur.122 PMC3076289

[B54] Shahbazian M. D. Antalffy B. Armstrong D. L. Zoghbi H. Y. (2002a). Insight into Rett syndrome: MeCP2 levels display tissue- and cell-specific differences and correlate with neuronal maturation. Hum. Mol. Genet. 11, 115–124 10.1093/hmg/11.2.115 11809720

[B55] Shahbazian M. D. Young J. Yuva-Paylor L. Spencer C. Antalffy B. Noebels J. (2002b). Mice with truncated MeCP2 recapitulate many Rett syndrome features and display hyperacetylation of histone H3. Neuron 35, 243–254 10.1016/S0896-6273(02)00768-7 12160743

[B56] Skene P. J. Illingworth R. S. Webb S. Kerr A. R. W. James K. D. Turner D. J. (2010). Neuronal MeCP2 is expressed at near histone-octamer levels and globally alters the chromatin state. Mol. Cell 37, 457–468 10.1016/j.molcel.2010.01.030 20188665 PMC4338610

[B57] Schnorr S. J. Steenbergen P. J. Richardson M. K. Champagne D. L. (2012). Measuring thigmotaxis in larval zebrafish. Behav. Brain Res. 228, 367–374 10.1016/j.bbr.2011.12.016 22197677

[B58] Stearns N. A. Schaevitz L. R. Bowling H. Nag N. Berger U. V. Berger-Sweeney J. (2007). Behavioral and anatomical abnormalities in mecp2 mutant mice: a model for Rett syndrome. Neuroscience 146, 907–921 10.1016/j.neuroscience.2007.02.009 17383101

[B59] Steenbergen P. J. Richardson M. K. Champagne D. L. (2011). The use of the zebrafish model in stress research. Prog. Neuropsychopharmacol. Biol. Psychiatry 35, 1432–1451 10.1016/j.pnpbp.2010.10.010 20971150

[B60] Thirumalai V. Cline H. T. (2008). Endogenous dopamine suppresses initiation of swimming in prefeeding zebrafish larvae. J. Neurophysiol. 100, 1635–1648 10.1152/jn.90568.2008 18562547 PMC2544474

[B61] Westerfield M. (2000). The Zebrafish Book a Guide for the Laboratory Use of Zebrafish Danio (Brachydanio) rerio. 4th edn Eugene, OR: University of Oregon Press.

[B62] Wood S. J. Toth M. (2001). Molecular pathways of anxiety revealed by knockout mice. Mol. Neurobiol. 2, 101–119 11817214 10.1385/MN:23:2-3:101

[B63] Xi Y. Noble S. Ekker M. (2011). Modeling neurodegeneration in zebrafish. Curr. Neurol. Neurosci. Rep. 11, 274–282 10.1007/s11910-011-0182-2 21271309 PMC3075402

[B64] Yasui D. H. Peddada S. Bieda M. C. Vallero R. O. Hogart A. Nagarajan R. P. (2007). Integrated epigenomic analyses of neuronal MeCP2 reveal a role for long-range interaction with active genes. Proc. Natl. Acad. Sci. U.S.A. 104, 19416–19421 10.1073/pnas.0707442104 18042715 PMC2148304

[B65] Young J. I. Hong E. P. Castle J. C. Crespo-Barreto J. Bowman A. B. Rose M. F. (2005). Regulation of RNA splicing by the methylation-dependent transcriptional repressor methyl-CpG binding protein 2. Proc. Natl. Acad. Sci. U.S.A. 102, 17551–17558 10.1073/pnas.0507856102 16251272 PMC1266160

[B66] Zupanc G. K. H. (2008). Adult neurogenesis and neuronal regeneration in the brain of teleost fish. J. Physiol. Paris 102, 357–373 10.1016/j.jphysparis.2008.10.007 18984045

